# Life or Death? A Physiogenomic Approach to Understand Individual Variation in Responses to Hemorrhagic Shock

**DOI:** 10.2174/138920211797248574

**Published:** 2011-09

**Authors:** Harold G Klemcke, Bina Joe, Rajiv Rose, Kathy L Ryan

**Affiliations:** 1 U.S. Army Institute of Surgical Research, Fort Sam Houston, TX 78234, USA; 2 Physiological Genomics Laboratory, Department of Physiology and Pharmacology, University of Toledo College of Medicine, Toledo, OH 43614, USA

**Keywords:** Controlled hemorrhage, epigenetic, genes, hemorrhagic shock, inbred rats, QTL.

## Abstract

Severe hemorrhage due to trauma is a major cause of death throughout the world. It has often been observed that some victims are able to withstand hemorrhage better than others. For decades investigators have attempted to identify physiological mechanisms that distinguish survivors from nonsurvivors for the purpose of providing more informed therapies. As an alternative approach to address this issue, we have initiated a research program to identify genes and genetic mechanisms that contribute to this phenotype of survival time after controlled hemorrhage. From physiogenomic studies using inbred rat strains, we have demonstrated that this phenotype is a heritable quantitative trait, and is therefore a complex trait regulated by multiple genes. Our work continues to identify quantitative trait loci as well as potential epigenetic mechanisms that might influence survival time after severe hemorrhage. Our ultimate goal is to improve survival to traumatic hemorrhage and attendant shock *via* regulation of genetic mechanisms and to provide knowledge that will lead to genetically-informed personalized treatments.

Throughout the world, traumatic injury is a major cause of death and the leading cause of years-of-life lost [1]. World-wide, hemorrhage is responsible for 30-40% of all traumatic deaths [1] and approximately 50% of battlefield deaths [2]. While many combat deaths are so severe that even immediate treatment using current medical capabilities could not save the victim, ~ 24% of combat deaths could potentially be prevented if medical treatment was immediately available; 85% of these potentially preventable deaths are due to severe hemorrhage [3].

In response to hemorrhage (either alone or combined with traumatic injury to tissues), a variety of physiological mechanisms are marshaled to maintain blood pressure and thereby tissue perfusion for as long as possible. When these compensatory mechanisms fail and tissue perfusion is diminished to critical levels, a condition of shock exists, defined as “an abnormality of the circulatory system that results in inadequate organ perfusion and tissue oxygenation” [4]. This can subsequently produce cellular dysfunction that may become irreversible without adequate resuscitation. Finding solutions to the continuing problem of severe traumatic hemorrhage and attendant shock is an ongoing objective of the U.S. military and many medical universities throughout the world, with most research focusing on development of procedures to stop bleeding and restore tissue perfusion [5-12]. Most such procedures have provided some, but not complete, reversal of adverse conditions associated with hemorrhagic shock, especially with very severe combat-related injuries. As an alternative approach, recent investigations have suggested that optimization of the ability of the tissues to withstand hemorrhage is critical and that the magnitude of this ability differs among individuals, thereby suggesting a genetic component. The purpose of this review is to summarize this recent work. 

## ALTERNATIVE APPROACHES

Bellamy and colleagues suggested in 1996 that: “… We need a new approach toward managing the rapidly exsanguinating casualty. We need to be able to pharmacologically induce a state of temporary tolerance to ischemia...” [13]. Other investigators stated that “…the key to improved care may not involve hemodynamics alone but likely requires an understanding of the molecular and cellular responses that are triggered by massive blood loss and shock. …Mechanisms that may induce tolerance to ischemia and cellular hypoxia (e.g., hibernation, ischemic preconditioning, and hypothermia) should be explored….” [14]. In agreement with these concepts, numerous studies have been conducted that seek to improve the ability of the organism to withstand the global ischemia of hemorrhagic shock when it is not possible to stop bleeding and restore normal perfusion (Table **1**). Most such approaches have been used primarily in animal models, but a few have been either used in humans (artificial hibernation, naloxone), or are awaiting clinical trials (hypothermia, glutamine; http://clinicaltrials.gov). Investigation of these and alternative compounds is currently an area of very active research.

## SURVIVORS *VS* NON-SURVIVORS

It has been noted for decades, however, that there is considerable variability in the ability of patients to survive traumatic hemorrhage [15]. Indeed, phenotypic variability is a commonplace occurrence in the realm of biology [16]. For humans, the source of this variability in the ability to survive severe hemorrhage is multifactorial and may include variation in the nature of the traumatic hemorrhage, the biological nature of victims (e.g., age, lifestyle, general health, chronic disease states), and the immediacy of trained caregivers and hospital facilities. If the innate ability of the individual to survive hemorrhage is considered as a phenotype of that individual, then it also becomes apparent that this phenotype can be influenced by the underlying genetic background of the individual.

Numerous studies have been conducted to characterize and understand the physiological differences between individuals that survive hemorrhagic shock and those that do not. Shoemaker and colleagues initiated studies as early as 1970 in attempts to understand the physiological differences between survivors and nonsurvivors of hemorrhagic shock [17]. Subsequent work by these investigators with trauma patients indicated that the physiological measurements most different between the two groups were cardiac index, oxygen delivery, and tissue oxygen utilization (e.g., [18-20]). This differing ability to deliver oxygen and use it at the tissue level is reflected in an association between lower blood lactate levels and an increased probability of survival [21]. Other studies have shown associations between patient survival and inflammatory cytokines [22] or heat shock proteins [23]. In rat studies, enhanced survival has been associated with higher respiration rates, arterial P_O2,_ hemoglobin oxygen saturation percentages, blood O_2_ content, and blood pH, as well as lower lactate levels [24-26]. All such measures and observations reflect the above-noted activation of multiple compensatory systems subsequent to severe hemorrhage, and provide clues to mechanisms associated with differential survival to hemorrhage. However, such clues are often difficult to interpret and prioritize importance as they represent the net effect of multiple mechanisms. 

## DETERMINING CELLULAR MECHANISMS

From the above brief review, it is obvious that factors contributing to differential survival are numerous and diverse in character, involving multiple systems, organs, tissues, and cellular pathways. Many different approaches could be and have been used in attempts to ascertain which cellular factors are both involved and important (e.g., [27 , 28-34]). Hence, there is a reasonably broad literature that indicates potential candidate genes involved in providing either: 1) the basal functioning from which each individual responds to hemorrhage; or, 2) the multiple and initially integrated responses to severe hemorrhage as the organism attempts to maintain homeostasis. All such procedures involve measurement of gene expression either at the mRNA or protein level. However, interpretation of gene expression (mRNA, protein, activity) for initial "discovery" procedures is confounded by the fact that expression is usually dependent on the types of tissue or cells used, and may also be dependent on the time of sampling. Therefore, sampling of multiple tissues at multiple times is often required to obtain an accurate assessment of gene expression.

## POTENTIAL GENETIC INVOLVEMENT

So the question becomes, “How should all such gene expression data be meaningfully integrated so that mechanisms that improve survival to hemorrhagic shock can be identified and ultimately manipulated?” The insightful thoughts contained within two salient articles that embodied years of research in the areas of cardiovascular function, blood pressure regulation, and hypertension provided potential answers [35-36]. This work focused our attention on the probability that variability in survival time (phenotypic variability) could be accounted for by genetic variability (polymorphisms), and hence that a genomic approach to solving this phenomenon might be both logical and appropriate. At the gene sequence level, single nucleotide polymorphisms (SNP) represent the smallest and most common element of variability accounting for phenotypic variability among individuals [37]. Epigenetic alterations at the gene level (detailed below) may also represent an important and widespread component of inter-individual variability [38]. While other investigators have previously explored gene expression and genetic polymorphisms in cells taken from trauma patients [39,40], systematic laboratory determination of the genes responsible for an organism’s ability to survive hemorrhage have not been previously undertaken. 

All characteristics of an organism are determined by that organism's DNA sequence and the interaction of that DNA sequence, transcripts, and proteins with each other and with the environment. The multiplicity and complexity of the many interacting mechanisms that ultimately lead to a given phenotype has been the subject of many recent reviews (e.g., [41-45]), and our knowledge in these areas is ever-evolving. Complex traits are those that do not exhibit classical Mendelian dominant or recessive inheritance associated with a single gene [46]. Rather, most depend upon the actions of multiple genes. These polygenic traits may be classified as either discrete or at least discontinuous [46,47] (i.e., cleft lip or palate, [48]) or as quantitative traits that are measured by continuous variables such as height and blood pressure [35, 46,47]. The gene loci or chromosomal regions that control a quantitative trait have been called quantitative trait loci (QTL), which are often broad chromosomal regions containing multiple genes, one or more of which is involved in controlling the phenotype of interest [35]. Most QTL span very large chromosomal regions (10-30 centimorgans, cM; in mice and rats, 1 cM is approximately equal to ~2,000,000 base pairs or 40 genes per cM). Hence, initial QTL may contain hundreds of genes, and subsequently need to be better defined [35,36]. 

The complexity of the response to hemorrhage and the multitude of factors potentially regulating this response suggest the probability that survival time after hemorrhage (STaH) may constitute a complex trait, and indeed a quantitative trait that demonstrates continuous variation. Moreover, if STaH is regulated by genes, then identification of these genes and their polymorphisms should ultimately provide not only an understanding of variant response mechanisms, but also the means to improve survival in victims of hemorrhagic shock. 

## A PHYSIOGENOMIC APPROACH

Strategies for determining the causal relationships between genes and complex physiological traits have been evolving and improving during the past two decades [49]. Indeed, multiple complex traits such as hypertension [35], stroke [50], obesity [51], aging [52], and type 2 diabetes [53] have received considerable investigatory attention as scientists and clinicians attempt to understand and ultimately regulate these traits. Having adopted such an approach, one may utilize a variety of tools and procedures for which there is ample evidence and documentation [54-59]. All such procedures are aimed at identifying variability in the sequence of nucleotides (polymorphisms) that might lead to differences in the amount or activity of proteins for which they code. Similarly, our approach is to gain knowledge that ultimately will improve patient care by being able to identify in each patient genetic and epigenetic markers that will allow for better-informed individual treatment. In this, our approach is both alternative and complementary to the efforts of other investigators seeking to find genetic mechanisms associated with enhanced survival to hemorrhage (see below).

**Fig. (1):**
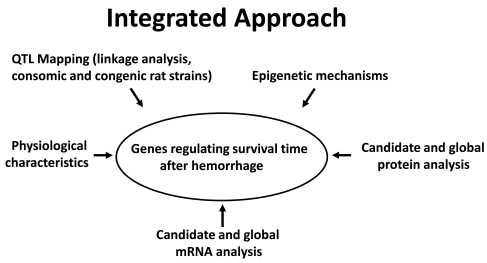
An integrated physiogenomics approach to identification of genetic polymorphisms and other genetic mechanisms that regulate survival time after severe hemorrhagic shock.

Our physiological genomic approach has initially focused on the identification of QTL, and ultimately genetic polymorphisms, associated with STaH in inbred rat strains (Fig. **1**). Additional genomic and molecular biological “tools” are being used to supplement the genomic-based approach. As these measures (mRNA, protein, and physiological characteristics) reflect gene expression, we remain mindful of the above-noted caveats in their interpretation; i.e., measured levels of such variables may be dependent on the time of measurement with reference to the hemorrhage, as well as on the site (tissue) of measurement. While hemorrhagic shock is accompanied by tissue damage that is often severe in trauma patients, we have focused only on the hemorrhage aspect of traumatic shock in our initial work. Rats are surgically catheterized, and 24 hours later are subjected to a severe hemorrhage while conscious and unrestrained. This experimental model therefore allows us to conduct studies that are minimally confounded by effects of anesthetics and analgesics.

**Fig. (2):**
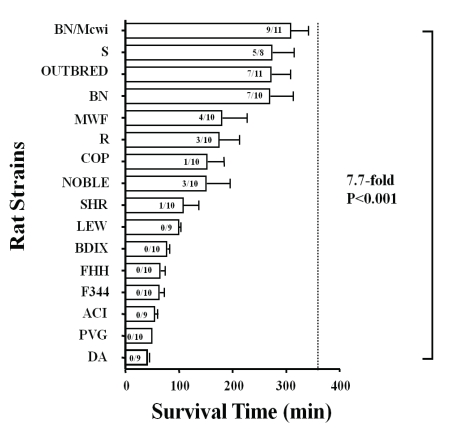
Survival time in inbred rat strains to a controlled hemorrhage. Values inside bars indicate the number of rats that lived / total number of rats for that strain. Bars represent the mean ± SEM. The vertical dashed line represents 360 min, the maximum observation period at which rats were euthanized if still alive (censored data). Rat strains: Brown Norway Medical College of Wisconsin (BN/Mcwi), Dahl Salt-Sensitive (S), Brown Norway (BN), Outbred Sprague Dawley (Outbred), Copenhagen 2331 (COP), Dahl Salt-Resistant (R), Lewis (Lew), Munich Wistar Fromter (MWF), BDIX, Spontaneously Hypertensive (SHR), Noble, Fawn Hooded Hypertensive (FHH), Black agouti (ACI), PVG, FISCHER 344 (F344), and Dark Agouti (DA) [33]. Reproduced with permission from Lippincott, Williams, & Wilkins.

As an initial attempt to identify genes associated with STaH, we tested the hypothesis that survival time after controlled hemorrhage is a heritable quantitative trait. To do this, we measured this phenotype (STaH) in multiple strains of inbred rats; individuals within inbred strains are genetically similar. Our data (Fig. **2**) demonstrated an apparent 8-fold difference in average survival time among strains [60]. Dark Agouti (DA) rats had the shortest average survival time (40 ± 5 min), whereas Brown Norway/ Medical College of Wisconsin (BN/Mcwi) had the longest survival time (306 ± 36 min). The percent of animals surviving the hemorrhage varied from 0 to 82% across strains (P < 0.001). 

**Fig. (3):**
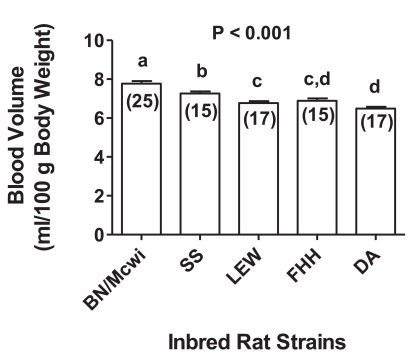
Normalized blood volume in inbred rat strains measured using an Evans Blue procedure. Each bar represents the mean ± SEM of the number of rats noted in parentheses within bars. Bars with different letter superscripts (a, b, c, d) are significantly different (P<0.05) [64]. Adapted from Physiological Genomics 43: 758-765, 2011; “Am Physiol Soc, used with permission”.

In this initial study, 55% of the calculated blood volume was removed from all rats using a blood volume to body weight ratio of 5.83 ml/100g body weight (BW) that was based on the average of previously reported blood volumes from outbred rats [61-63]. In designing this first study, we accepted the fact that this ratio may differ among inbred rat strains, and we considered such differences to be one of the genomic-associated mechanisms that might be related to differences in STaH. We therefore chose five strains for use in studies designed to address concerns that blood volumes might be different among these inbred rats and to ensure that future measurements of survival time reflected the same hemorrhagic challenge. The choice of these five strains was based on their differences in survival time and on the availability of genetic “tools” (i.e., consomic and congenic rat strains) for subsequent studies. These inbred strains were: BN/Mcwi (the strain with the longest STaH), Fawn Hooded Hypertensive (FHH), Dahl Salt Sensitive (SS), Lewis (LEW), and DA rats (the strain with the shortest STaH). We then measured blood volumes in rats from each inbred strain using an Evan’s Blue dye procedure. We found not only that blood volumes differed (Fig. **3**), but also that this trait had a high degree of heritability (h^2^ = 0.56) [64]. 

**Fig. (4):**
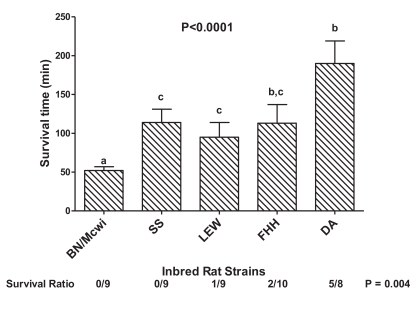
Survival time in inbred rat strains to a controlled 47% hemorrhage. Values below bars indicate the number of rats that lived / total number of rats for that strain. Bars represent the mean ± SEM. Rat strains: Brown Norway Medical College of Wisconsin (BN/Mcwi), Dahl Salt-Sensitive (SS), Lewis (Lew), Fawn Hooded Hypertensive (FHH), and Dark Agouti (DA). Overall results of strain comparisons for survival times (log-rank test) and for survival ratios (Chi-Square test) are presented. Bars with different letter superscripts (a, b, c, d) are significantly different (P<0.05) [64]. Adapted from Physiological Genomics 43:758-765, 2011; “Am Physiol Soc, used with permission”.

With this information, it appeared quite possible that differences in survival times measured in our initial study might in part reflect the different blood volumes inherent to each strain and therefore differences in percentage of blood removed. Hence, we repeated our initial study using the now known average normalized blood volumes to calculate the amount of blood removal required to achieve a 47% reduction in blood volume. We found that, with comparable degrees of hemorrhage, rats from these inbred rat strains responded differently (Fig. **4**) from the original study. Indeed, DA rats (now the longest survivor) lived on the average ~3-fold longer than the shortest lived survivor, BN/Mcwi [64]. Moreover, STaH appeared to be a heritable (h^2^ = 0.44) quantitative trait. From this study, we concluded that, while the inherent blood volume played a major role in determining STaH, other genetic factors were also involved in producing this phenotype.

In related attempts to determine genes associated with enhanced survival to traumatic hemorrhage, other investigators have focused on specific candidate gene polymorphisms to determine their association with mortality in trauma patients. Such investigations indicate that specific polymorphisms associated with mitochondrial Complex 1 [65] and complement component 2 [66] may be associated with increased mortality of trauma victims. Alternatively, a polymorphism in a β-2 adrenergic receptor (ADBR2: Q27E) was associated with enhanced survival after various blunt and penetrating injuries [67].

## COMPLEMENTARY RESEARCH

The focus of our short review is hemorrhagic shock resulting from injury and rapid exsanguination. Moreover, at least initially, our research focus is concerned with the initial time interval after injury (0-6 hours) during which hemorrhagic shock has the greatest impact on mortality [68]. There are, however, other clinical conditions resulting from trauma but not specifically related to hemorrhagic shock that have received more attention from a genetic and genomic approach. The thread that connects this research is the quest to understand and ultimately to treat the variability in individual responses to these trauma-associated conditions.

As noted above, other investigators have begun examining the complexity of organismal responses to trauma [39-40], and have aggressively focused their efforts on understanding inflammatory responses to trauma. A great deal of this work has been accomplished *via* the establishment of the *Inflammation and the Host Response to Injury* consortium, funded as a "glue" grant by the National Institute of General Medical Sciences (e.g., [69-72]).

Sepsis is a fairly common consequence of severe traumatic hemorrhage that results from an altered functioning of the immune system [73] that may ultimately lead to multiple organ failure [74]. Differences in inflammation-related genes associated with differential susceptibility to sepsis have been recently reviewed [75,76]. Most work to date has focused on polymorphisms of candidate genes associated with differing inflammatory responses that result in differing individual susceptibilities to sepsis.

Research into a genetic involvement in variability in responses to similar traumatic brain injuries has also been conducted for over a decade [77,78]. Much of this genetic work has involved polymorphisms of apolipoprotein E [77-79] but polymorphisms of multiple genes have also come under scrutiny [80]. To date, all such physiogenomic-based studies have investigated candidate gene polymorphisms almost exclusively in human patients [80].

Finally, severe traumatic hemorrhage often makes victims more susceptible to acute lung injury (ALI) [81]. ALI has been studied at the genetic and genomic levels using candidate gene association studies [82] as well as genome wide linkage analyses [83]. Although hyperoxic toxicity rather than traumatic injury was used to induce ALI, genetic determinants of, and differing susceptibilities to, ALI were first indicated in mice [84]. Subsequently, numerous candidate genes have been identified that are associated with multiple underlying causes of ALI [82, 85].

## FUTURE RESEARCH APPROACHES

Overall, our investigations conducted using rat models provide compelling evidence to suggest that STaH is a quantitative trait. The ultimate resolution of genetic factors contributing to this trait are expected to be at the level of a SNP or a select combination of a group of SNPs (called a haplotype block). The challenge that lies ahead is to identify loci that control the quantitative trait of STaH. How does one proceed? Mere interpretations of comparisons at the physiological and biochemical levels of two inbred strains is conceptually flawed because of the inability of such comparisons to discern whether observed changes at these levels are: a) the cause of STaH differences; b) the consequence of STaH differences; or, c) due to genetic drift (chance selection and fixation of genetic differences unrelated to STaH that are forced to occur during inbreeding) [86-88]. The solution to this problem is to seek an experimental design that avoids the bias of inferences drawn by biochemical or physiological functions elicited by the action of any given locus (QTL). Such a design–called a linkage analysis– uses genetically segregating populations derived by breeding two strains of rats and phenotyping them for STaH. Such an experimental design allows for locating QTLs solely based on their position on the genome [35]. The results of a linkage analysis are corroborated by substitution mapping using congenic strains [89]. These steps are depicted in Fig. (**5**) and detailed below. 

### Stage 1: Genetic Linkage Analysis

The first step is to establish statistically significant genome-wide evidence for linkage of large regions of the genome to STaH. To do so, the two inbred rat strains most divergent in STaH (DA females and BN/Mcwi males) will be bred to generate a first filial (F_1_) generation. F_1_ rats are identical, but heterozygous having received one copy of each chromosome from each parent. The male F_1_ will be phenotyped and their phenotype compared with the parental strains. There are multiple potential outcomes for the F_1 _phenotype especially as the number of loci involved with STaH increase [90]. If the purpose of the research is to detect and to identify one or more major loci that have dominant effects on the phenotype of interest (in our case STaH), and if F_1_ rats have an average STaH that is close to that of one of the parental strains, then it is most efficient to conduct a backcross of F_1_ individuals to the opposite parental strain to produce the N_2_ generation [90,91]. On the contrary, the F_1_ STaH might lie mid-way between that of the two parental strains suggesting the absence of dominance of any loci and the presence of additive effects. With such results it would be more efficient to conduct an intercross of F_1_ individuals to produce an F_2_ generation [91] . Subsequently, representatives (~ 200 rats) of either the N_2_ or the F_2_ will be phenotyped (STaH measured) and genotyped, and QTL will be identified. Genotyping will be conducted using Affymetrix GeneChip rat Mapping 10 K Single nucleotide polymorphism (SNP) arrays to achieve a genome-wide scan. Using this approach, information on chromosomal location, the magnitude of the STaH effect, and the mode of inheritance of the causative genes can be obtained. 

### Stage 2: Substitution Mapping

**Fig. (5):**
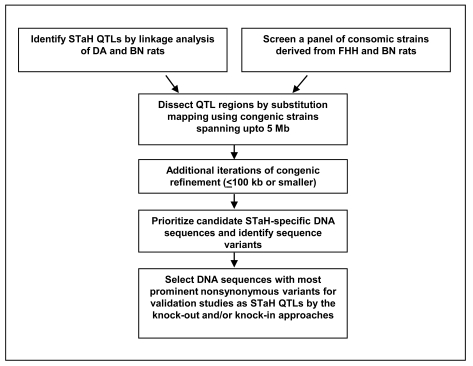
Diagrammatic representation of our approach to identify DNA sequences influencing survival time after severe hemorrhage (STaH) in inbred rat strains. Such DNA sequences may include those that are transcribed into mRNA that will be translated into proteins, and sequences transcribed as non-coding RNAs such as microRNAs, small interfering RNA, and long non-coding RNA [173]. Dark Agouti (DA); Brown Norway/ Medical College of Wisconsin (BN); Fawn Hooded Hypertensive (FHH); Quantitative Trait Loci (QTL).

Linkage analysis results in the identification of genomic regions of approximately 5 to 30 Mb. A genetic interval of this size typically corresponds in humans and rodents to ~50 to 300 genes [92], which is far too many positional candidates to begin functional evaluation (i.e., a determination of the gene product and its function) of each individual gene. Thus, initial low-resolution linkage studies typically establish the map location to a resolution that is sufficiently precise to confirm and define the genomic interval. In model systems, this can be done with the use of consomic strains [93], congenic strains or near-isogenic lines (e.g., [94-96]). The most popular among these methods that is successfully adapted in rodent models is to use congenic strains (Fig. **5**), which was first described by Snell [97]. The basic technique (as detailed by Cowley *et al*. [98]) involves substituting a segment of chromosome from one strain (the donor strain) into another strain (the recipient strain). This is done by crossing the donor and recipient strains to produce F_1_ animals, and then crossing F_1_ x recipient (first backcross). The offspring of this first backcross are genotyped using tail biopsy DNA for markers across a putative QTL-containing region of a chromosome. Because chromosomal crossovers will occur between the donor and recipient chromosomes in meioses of F_1_ animals, recombinant chromosomes will be found in some offspring. This means that an animal with a specified donor chromosomal segment can easily be found. Such offspring are backcrossed again to the recipient, and offspring carrying the specific donor chromosomal segment are again selected. This procedure is repeated at least 8 times. At each backcross, half the unlinked heterozygous loci outside the selected (congenic) region become homozygous for the recipient allele; that is, the genetic background progressively becomes that of the recipient strain. After these ~ 8 backcrosses to the recipient strain, rats are selectively bred to fix the donor chromosomal segment in the homozygous state on the background of the recipient strain, producing a congenic strain. The expectation is that the introgressed donor segment will alter the phenotype of the recipient strain. Because the construction of congenic strains relies on recombinations, as long as the phenotypic change is traceable, it is second to none as a method for finding genes. Occasional disdain toward using congenic strains that is found in the literature [99] is not due to the reliability of the technique itself, but due to the fact that it is a laborious and time consuming procedure. Consomic strains are generated the same way as congenic strains except that entire chromosomes instead of chromosomal segments are substituted.

Post-linkage analysis, congenic strains have also been constructed and studied for a variety of traits in rat models of diseases such as obesity [100], arthritis [101-102], hypertension [103-107] insulin resistance [108] and other phenotypic traits [109]. We have demonstrated success at constructing congenic strains and ‘trapping’ (i.e., introgressing a specific chromosome segment from one inbred rat strain to the genome of a second inbred rat strain) QTL for different disease models, including rheumatoid arthritis [101-102], albuminuria [110] and high blood pressure [55, 111-113]. Similar to such studies, congenic strains will be constructed to ‘trap’ STaH QTLs located using DA and BN/Mcwi rats. Alternately, consomic panels available for FHH and BN/Mcwi rats are also useful for mapping STaH QTLs (Fig. **5**). 

### Stage 3: Fine Substitution Mapping

The next step, regardless of the consomic or congenic approach, is to reduce the size of the critical interval containing the QTL as much as possible (Fig. **5**). This serves to reduce the size of the candidate interval sufficiently such that the number of candidate genes is modest and functional studies can be undertaken. These approaches may be used to reduce the minimal interval to less than 1 Mb. This will be done by backcrossing the parental congenic strain with one of the inbred strains. Subsequent intercrosses ensure the homozygosity of the congenic substrain [114]. 

In parallel with the conduct of linkage analyses and substitution mapping procedures, we will also continuously evaluate candidate genes for polymorphisms. The challenge, of course, is to choose candidates that have the highest probability of being related to our phenotype (STaH). Our approach involves multiple means by which to address this challenge. First, candidate genes will be identified from the literature as those associated with closely related phenotypes such as cardiac ischemia, acute lung injury, stroke, traumatic brain injury. Second, genes whose mRNA or protein expressions are significantly different among inbred rat strains divergent in STaH will be evaluated as potential candidate genes. Third, using information obtained in steps 1 and 2, an in-silico analysis will be conducted using SNPlotyper [115] to identify polymorphisms associated with, or in very close proximity to, those candidate genes. Finally, we will screen chosen polymorphic genes for SNPs among rats from our survival studies. Such procedures will supplement ongoing linkage analyses and have the potential for expediting gene discovery. Moreover, they provide information that eventually may be used to eliminate putative candidate genes after narrowing down loci using mapping studies. 

### Stage 4: Search for Sequence Variants

Minimal QTL intervals (the smallest practical chromosomal segment that can be introgressed into congenic strains based on naturally occurring meiotic recombinations) of <100 kb ‘trapped’ within congenic strains often include more than a single gene [112, 116-117]. The fourth and final step is to determine the DNA sequences within the minimal QTL interval that are different between BN/Mcwi and DA. Advancement in rapid DNA sequencing technologies such as the Nextgen sequencing methods [118] is highly favorable for obtaining comprehensive data on the candidate variants accounting for a particular QTL. Some QTLs result from single nucleotide substitutions, while others result from several variant nucleotides. As a result, each candidate nucleotide variant as well as all combinations of candidate nucleotides in one or several genes must be identified, prioritized, and functionally tested. In some cases, it is likely that such variants may not reside within protein-coding genes. For example, we recently mapped a blood pressure QTL to a <81.8 kb congenic segment with no known protein-coding genes [119]. At this stage, one could utilize complementary evidence resulting from studies measuring gene expression (mRNA and protein) to accumulate compelling evidence for one gene/non-coding genetic element being the most relevant in this minimal QTL interval. Indeed, the potential importance of non-coding RNAs in the overall response to hemorrhagic shock has been suggested by changes in non-coding RNAs in various cell types in response to ischemia and traumatic brain injury [120-124]. Depending on the nature of this superior causative candidate gene, other approaches such as constructing transgenic rats and/or using technology such as silencers of mRNA (RNAi or siRNA) could be used. The former has been successfully applied to prove that the *Cd36* gene is a causative factor for insulin resistance in SHR rats [125,126].

### Stage 5: Validation Studies

The congenic method has a limitation of not being able to resolve regions beyond what is permissible by natural recombinations. The extent of resolution achieved can be under 100 kb but all variants within this short segment remain as candidate genetic determinants of STaH. Even if it was possible to narrow the QTL interval further *via* recombination, it is highly unlikely that it will be comprised of only a single candidate gene. Rather, additional sequences of nucleotides –that may represent other genes– will undoubtedly be on either side of the candidate gene of interest. Hence, functional studies to validate the importance of the candidate gene for our phenotype (STaH) need to be conducted. Such studies are most effectively conducted using genetically-engineered models such as loss-of-function (knock-out) or gene replacement (knock-in) rats.

### Genetically-Engineered Rat Models

In recent years, the big impediment of not being able to generate targeted gene disruptions or ‘knockouts’ in rats has been largely alleviated. The use of homologous recombination to modify genes in embryonic stem (ES) cells was recently described in rats as a powerful means to elucidate gene function [127]. Another, more popular method, which was first described in zebrafish [128-130] and later in rats [131], is the zinc-finger nuclease (ZFN) based targeted gene-disruption. Both of these knock-out methods are highly specific to the locus in question [132], unlike non-specific gene effects that are to be expected from the transgenic approaches wherein a foreign DNA is inserted and could potentially result in multiple gene integration sites on the genome. Such results would lead to multiple gene-gene interactions that might obscure the actual function of the transferred gene. The feasibility of the ES cell based homologous recombination and ZFN-based knockout approaches in rats were demonstrated by the construction and characterization of p53 (a tumor suppressor) [127] and renin [56] gene knockout rats, respectively .

Therefore, validation studies of STaH candidate genes can be attempted using gene knockout rats. Alternatively, a recent report describes the feasibility of a ‘knock-in’ approach in rats wherein a select sequence-of-choice is introduced into the nuclear genome by homologous recombination [133]. This emerging approach will be an additional valuable tool for providing proof-of-principle for a candidate gene variant to be assessed as a cause for observed differences in STaH between DA and BN/Mcwi rats. The successful completion of all the above stages results in the discovery of a positionally cloned novel gene that contributes to STaH. 

### What is the Translational Significance?

In the context of hemorrhage, any genes contributing to increasing STaH that are identified in rats would serve as strong candidate genes to evaluate in human victims of hemorrhagic shock. Using this population, screening for polymorphisms of these genes would determine whether there are associations of these genes with survival to hemorrhagic shock. Indeed, positional cloning projects for complex traits in rats are most rewarding when parallel observations of candidate gene associations, or related pathways, are reported for the same complex trait in humans. For some of the most advanced mapping studies in hypertension, such parallel associations have been demonstrated in both species. For example, positional cloning approaches in rats identified that loss of the Fc gamma receptor 3 (Fcgr3) gene is a determinant of macrophage overactivity and glomerulonephritis in Wistar Kyoto rats [134]. In humans, low copy number of FCGR3B, an orthologue of rat Fcgr3, was associated with glomerulonephritis in the autoimmune disease systemic lupus erythematosus [134,135]. Such parallel observations are likely to increase in future years because of the advent of dense mapping studies of large human populations (n>1000) through genome-wide association studies. In the likelihood that association studies are not available for the positionally-cloned gene for STaH, associations can be sought through custom-designed association studies of DNA repositories of individuals with pre-recorded phenotypic data on STaH. There is at least one such association study reported in the field of hypertension. A Disintegrin-like MetalloProteinase with Thrombospondin motifs, 16 (*Adamts16*) was a gene positionally prioritized in rats, which was then tested for association in hypertensive versus normal subject cohorts [119]. *ADAMTS16* was indeed significantly associated with human hypertension in two independent cohorts [119]. 

## A POTENTIAL ROLE FOR EPIGENETICS IN RESPONSES TO HEMORRHAGIC SHOCK

**Fig. (6):**
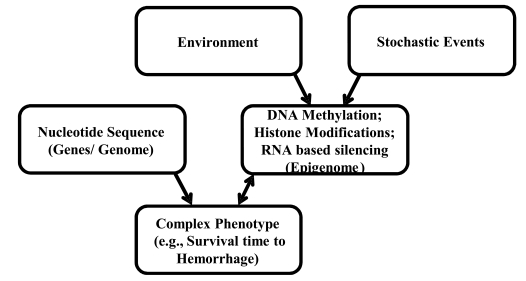
Not all complex phenotypes can be fully explained by genes, environment, or their interactions. Numerous environmental factors and stochastic (random) events alter epigenetic processes which may subsequently alter a phenotype. Certain complex phenotypes can also influence the epigenome (e.g., ischemic responses).

We also recognize that all phenotypic variability among individuals, even isogenic individuals, cannot be fully explained by classical genetics [136] and environment (Fig. **6**). Phenotypic variability among inbred animals has been attributed to a non-genetic and a non-environment “third component” [137,138] now known as epigenetic processes [139]. More recently, epigenetic processes have been shown to result in phenotypic differences among monozygotic twins [139-141] and inbred rat strains [142-145]. Numerous environmental factors such as maternal care [146], early-life stress [147], smoking [148], alcohol [149], air pollutants [150], exercise [151] and diet [152] can alter epigenetic processes to varying extents at both global and gene promoter levels.

Defined as heritable changes in gene expression not coded by the DNA sequence, major epigenetic mechanisms include DNA methylation and post-translational histone modifications [153]. They are critical for normal genomic functions and act to regulate gene expression. DNA methylation, histone modifications and their interplay are the most studied epigenetic mechanisms [154]. Epigenetic processes, especially DNA methylation, are more dynamic than DNA sequence changes and can be altered by environmental and non-environmental stochastic (random) events. Further, epigenetic changes may be site specific and may vary with tissues or cell types.

DNA methylation is the best characterized epigenetic process in mammals [155] and entails covalent addition of methyl (CH_3_) groups on cytosines situated in the cytosine-guanidine dinucleotide [156,157]. DNA methylation represses gene transcription while hypomethylation is associated with active transcription [158]. DNA methylation is essential for normal embryonic development and differentiation, genomic imprinting, X-chromosome inactivation, genome stability, and tissue specific gene expression [159,160]. Histone modifications are another type of epigenetic regulation, with acetylation being the best-understood of the many types of histone modifications known to exist [154]. Histone acetylation is a reversible modification of select residues in the histones and is regulated by histone acetylases and histone deacetylases. Histone acetylation can occur globally or at specific promoters [154]. Acetylation marks transcriptionally active regions while deacetylated histones are found in transcriptionally repressed regions. 

In addition to involvement with a number of human diseases [161], epigenetic processes have been demonstrated in various animal models in response to local [162-165] and global ischemia (e.g., [166]), as well as with the hypoxia that attends such conditions [167]. Several studies have now shown that alteration of histone acetylation profiles *via* manipulation of histone deacetylase inhibitors improves survival to an otherwise lethal hemorrhage [166, 168,169]. However, an important question that remains unresolved is whether specific epigenetic patterns are associated with responses to hemorrhagic shock. 

We have observed considerable intra-strain variation in the STaH in our studies, despite the use of inbred rat strains raised in very similar environmental conditions and with highly reproducible experimental conditions [64]. We now suspect that epigenetic mechanisms may be partly responsible for such variability. Hence, both genetic and epigenetic mechanisms may be predisposing factors for STaH, and our approach (Fig. **1**) now includes efforts to identify epigenetic mechanisms. Indeed, a similar speculation has previously been made based on the extensive variability in survival ability to hypoxia in inbred mice subjected to whole body hypoxic pre-conditioning [144]. 

## SUMMARY

At this time in the evolution of attempts to first identify, and then to regulate, genetic mechanisms associated with responses to hemorrhagic shock, we are at an early discovery stage. A challenge is to meaningfully integrate the large amounts of data that will be forthcoming. The realm of systems biology [170] and the rapidly maturing tools of bioinformatics should assist. We understand that our approach is unlikely to discover all such mechanisms associated with STaH, but rather will focus on those genes that are different among the inbred rat strains to be studied. Other gene combinations and interactions might be operable in different inbred rat strains to achieve improved survival. However, our ultimate objective is not to determine all genes involved, but only ones that are important enough such that their manipulation will improve survival to hemorrhagic shock. 

The epigenome represents epigenetic patterns on a genome-wide basis [171]. The relationship between the genome and the epigenome is as yet unclear. For example, it may be possible that the epigenome and its response to environmental factors may be determined by the genotype. What is clear is that the presence of an epigenome, and the fact that different cell types contain different epigenomes, adds additional layers of complexity and difficulty to the process of associating phenotypes with genotypes for complex quantitative traits. Indeed, it is quite likely that both genetic and epigenetic variability interact to influence phenotypic variability [172]. Advances in high throughput screening techniques will facilitate identification of both genome-wide and cell-specific epigenomic patterns. An improved understanding of the role of both genetics and epigenetics in contributing to this complex quantitative trait, STaH, will be of importance in the development of novel biomarkers for evaluation of varying degrees of susceptibility, and for development of future individual-specific therapeutics (i.e, personalized medicine).

## Figures and Tables

**Table 1:** Some Alternative Approaches Tested to Improve Survival to Hemorrhagic Shock

Approach	Representative References	Translated to Clinical Trials or Patient Use for Hemorrhagic Shock
Artificial Hibernation	[174-176]	Yes
Hypothermia	[177-179]	Yes
Hydrogen Sulfide	[180-182]	No
Melatonin	[183-185]	No
Β-hydroxybutyrate	[186-188]	No
Ethyl Pyruvate	[189-190]	No
Estradiol	[191-193]	No
Crocetin	[194-196]	No
Glutamine	[197-199]	Yes
Histone deacetylase inhibitors (e.g., valproic acid)	[168-169, 200-201]	No
Naloxone	[202]	Yes
